# The Impact of Cardiovascular Antecedents on the Prognosis of COVID-19 Critically Ill Patients

**DOI:** 10.3390/jcm13123518

**Published:** 2024-06-15

**Authors:** Luiza Camelia Nechita, Mariana Daniela Ignat, Alexia Anastasia Stefania Balta, Raisa Eloise Barbu, Liliana Baroiu, Doina Carina Voinescu, Aurel Nechita, Mihaela Debita, Camelia Busila, Ioana Anca Stefanopol

**Affiliations:** 1 Doctoral School of Biomedical Sciences, ‘Dunarea de Jos’ University, 800008 Galati, Romania; nechitaluiza2012@yahoo.com (L.C.N.); alexiaanastasia1998@yahoo.com (A.A.S.B.); raisauibariu@gmail.com (R.E.B.); 2 Clinical Medical Department, Faculty of Medicine and Pharmacy, ‘Dunarea de Jos’ University, 800008 Galati, Romania; lilibaroiu@yahoo.com (L.B.); carinavoinescu@gmail.com (D.C.V.); nechitaaurel@yahoo.com (A.N.); camelia_busila@yahoo.com (C.B.); 3 ‘Sf. Cuv. Parascheva’ Clinical Hospital of Infectious Diseases, 800179 Galati, Romania; debita_mihaela@yahoo.com; 4 ‘Sf. Apostol Andrei’ Clinical Emergency County Hospital, 800578 Galati, Romania; 5 ‘Sf. Ioan’ Clinical Hospital for Children, 800487 Galati, Romania; ancaflorea1969@yahoo.com; 6 Medical Department, Faculty of Medicine and Pharmacy, ‘Dunarea de Jos’ University, 800008 Galati, Romania; 7 Clinical Surgical Department, Faculty of Medicine and Pharmacy, ‘Dunarea de Jos’ University, 800008 Galati, Romania

**Keywords:** cardiovascular disease, mechanical ventilation, COVID-19

## Abstract

**Background/Objectives**: The objective of the study is to analyze the impact of cardiovascular history on mortality in COVID-19 patients, hospitalized in the intensive care unit with indications for continuous positive airway pressure (CPAP) and subsequently mechanical ventilation, without oncological disease. **Methods**: A retrospective observational study was carried out on a group of 108 critical COVID-19 patients. We compared demographic data, paraclinical and clinical parameters, days of hospitalization, and mortality rate between two groups of patients, one group with a history of cardiovascular disease (81 patients) and a group without a history of cardiovascular disease (27 patients). **Results**: Patients with cardiovascular antecedents had a higher mortality rate than those without cardiovascular antecedents, presenting severe forms with shorter survival time in the intensive care unit and increased inflammatory evidence. Compared to patients without a history of cardiovascular illness, those with cardiovascular disease had a lower average age, and developed a severe form of COVID-19. **Conclusions**: Cardiovascular antecedents can worsen the prognosis of patients with COVID-19, requiring a careful screening and multidisciplinary approach.

## 1. Introduction

The COVID-19 pandemic has affected the entire population, resulting in, as of 704,753,890 illnesses and 7,010,681 deaths worldwide by 20 April 2024 [1]. Patients with multiple comorbidities, including heart conditions, were predisposed to severe forms of COVID-19 with a poor prognosis. Clinical studies have demonstrated that a history of heart failure predisposes to intra-COVID-19 myocardial damage [2,3] and the clinical manifestation of cardiovascular disease is an unfavorable prognostic factor in COVID-19 [4,5]. Understanding and objectifying the pathophysiological mechanisms of cardiac involvement in COVID-19 will be the basis for perfecting the management of patients with this impairment.

The aims of our study were to determine the impact of cardiac history on COVID-19 prognosis in critically ill patients, summarize the pathophysiological and anatomicalpathology evidence in the literature regarding COVID-19-induced cardiac damage, and compare the clinical outcomes regarding the impact of pre-existing cardiac damage on the evolution of COVID-19.

## 2. Materials and Methods

**Figure 1:**
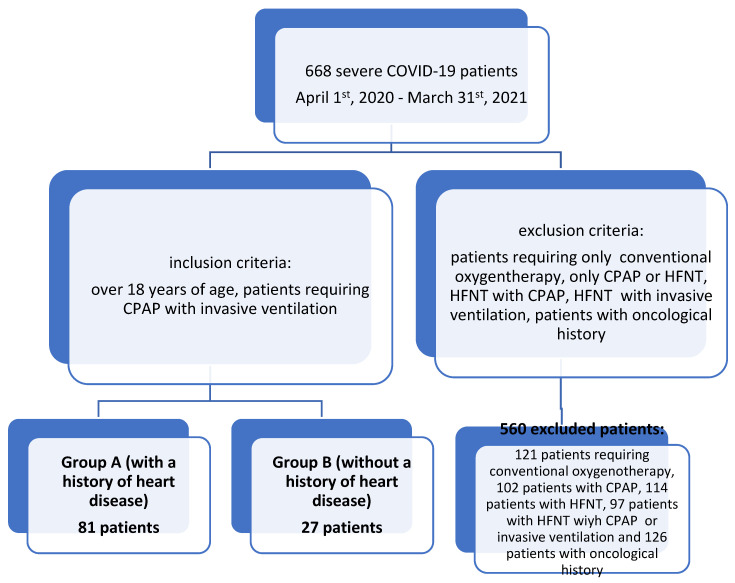
Patient group selection.

A comparative retrospective study was conducted on 668 adult patients with COVID-19, admitted to the ICU of the “Sfantul Apostol Andrei” Emergency Clinical Hospital of Galati, Romania, from 1 April 2020 to 31 March 2021. We analyzed 2 groups of patients with severe COVID-19 using CPAP and subsequently, invasive ventilation. The criteria were as follows: one group consisted of 81 patients with a history of heart disease and the second group comprised 27 patients without a history of heart disease before the onset of COVID-19 (Figure 1).

Patient inclusion criteria were represented by the patient’s COVID-19 status and their clinical characteristics—the severity of the disease, manifested as pneumonia, clinically and radiologically, usually complicated by respiratory failure or other complications such as thromboembolism, myocardial damage, and arrhythmias [6]. There were no specifics on demographic criteria (male or female patients, over 18 years of age). Patients from both groups presented acute respiratory failure associated with SARS-CoV-2 infection, hypoxemia (SpO2 < 90% under standard oxygen therapy), and moderately increased respiratory effort. CPAP selection criteria were patients with a low degree of anxiety and increased tolerability; an absence of pre-existing pathologies predisposing to the occurrence of pneumothax/pneumomediastinum during the use of positive pressure mechanical ventilation. The criteria for replacing CPAP with invasive ventilation were severe increased respiratory effort with the use of accessory muscles or respiratory frequency over 30 breaths/minute, risk of aspiration pneumonia, severe decompensated acidosis (pH < 7.2–7.25), and altered state of consciousness.

Exclusion criteria for the study consisted of pregnancy and lactation, as well as outliers (patients with extreme results in routine laboratory tests such as the total number of leukocytes, hemoglobin, erythrocyte sedimentation rate (ESR), urea, creatinine, fibrinogen, bilirubin, ferritine, and C-reactive protein). COVID-19 patients with recent surgical procedures, oncologic history or other non-respiratory illnesses requiring intensive care were also excluded. Patients who only required conventional oxygen therapy, only CPAP or HFNT, and those who required HFNT with CPAP or HFNT with invasive ventilation were excluded.

During hospitalization, the patients were monitored in the intensive care unit. Importantly, not all patients had complete data. The endpoint of the study was the total number of deaths.

The study was conducted according to the guidelines of the Declaration of Helsinki (following the STROBE guidelines) and approved by the Ethics Committee of “St. Apostol Andrei” Emergency Clinical Hospital of Galati, with the approval number 26872 on 3 December 2020. 

Patient data extracted from the observation sheets were analyzed using IBM SPSS Statistics 26 software. Gross descriptive statistical parameters were calculated for all variables, for which this analysis was useful. The presentation is in mean ± DS (standard deviation) for continuous variables and in absolute frequency (relative frequency) for categorical variables. The tests used in the inferential analysis were Student’s *t*-test, chi-squared test or χ2 test and charts for age groups, and were specified for each variable. 

Comparisons were made between the group of 81 patients with a history of heart disease and the second group of 27 patients without a history of heart disease before the onset of COVID-19. A level of statistical significance was considered for values below 0.05 (with two-tailed *p*-value) for each of the statistical tests used.

## 3. Results

Between 1 April 2020 and 31 March 2021, a total of 108 patients were admitted to “Sfantul Apostol Andrei’s” Emergency Clinical Hospital’s Intensive Care Unit (ICU), with severe COVID-19 using CPAP and subsequently, invasive ventilation criteria, with 37 (34.2%) survivors.

Two types of comparative analyses were conducted. The first analysis compared group A, consisting of patients with known cardiac pathologies (81 patients, 75%) and the other group is named group B, consisting of patients without cardiac comorbidities (27 patients, 25%).

The first analysis exposes the differences between the distribution of the patients between the two groups regarding the ordinal variables and some other variables of interest in the evolution of the patients (Table 1).

The comparative analysis presented the following conclusions for the present research:

- Regarding the sex of the patients in the study sample, the predominance of male subjects will be noted, regardless of the classification in the two study groups. This variable is one without statistical significance according to the chi-square test (*p* = 0.5).
- Regarding radiological features, the comparative analysis presents the following conclusions.
- Patients with pathological antecedents of cardiovascular diseases presented radiological changes such as lung opacities (58% versus 37%) and changes such as accentuated pulmonary interstitium (30.9% versus 25.9%), compared to patients without pathological antecedents of cardiovascular disease, which is statistically significant (*p* = 0.003).
- We specify that for three patients (11.1%) from group B the lung X-ray revealed pleurisy. Also, no radiological changes were observed in nine patients (11.1%), from group A and in seven patients (25.9%) from group B.

As stated earlier, the second statistical analysis compares the differences in distribution between a series of scalar variables, and patients belonging to one of the two groups mentioned above. In this type of analysis, the *p*-values corresponding to the T-tests performed in order to assess the degree of statistical significance will be found in the summary tables (Table 2).

**Figure 2:**
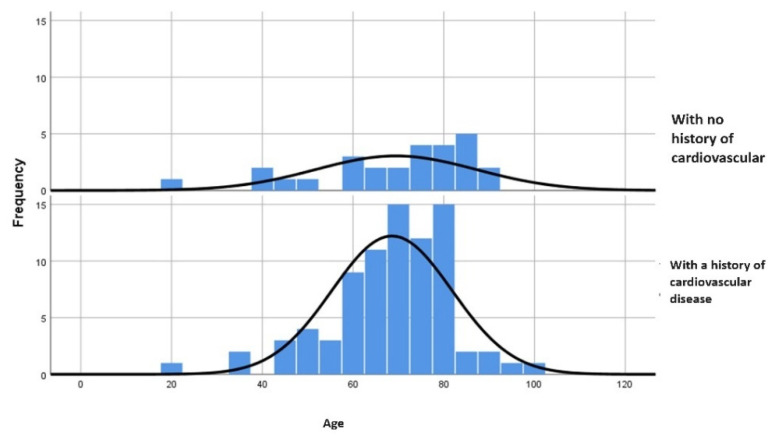
Comparison of ages between the two study groups.

The mean age of subjects in group B (69.44 years) is higher compared to group A (68.57 years). The maximum extreme value is higher in those who associate with cardiac pathology. The *p*-value of <0.001 emphasizes the existence of statistical significance associated with this test (Figure 2).

The oxygen saturation of the patients upon admission to the ICU was between 48% and 90%, without statistically significant differences between the two groups.

The duration of hospitalization was statistically significantly longer in group B (4.98 versus 7.78) (*p* < 0.001). This can be interpreted in the context in which the shorter duration of hospitalization may be correlated with the higher severity of forms of COVID-19 in patients with a history of cardiovascular disease and with the higher percentage of deaths among them. 

Paraclinical values expressed in scalar variables were analyzed comparatively, corresponding to previous models. We noted the following differences:

The mean total leukocyte count was lower in group A compared to group B, without statistical significance.

Regarding the inflammatory syndrome, the average C-reactive protein in group A was statistically significantly higher, compared to group B, with *p* < 0.001. Also, ESR has a higher mean in group A than in group B, without a statistically significant difference.

The mean LDH in group A was statistically significantly higher than that of group B, *p* = 0.022. Regarding the procoagulant pattern of COVID-19, the mean value of D-dimers was higher in group A, but without a statistically significant difference compared to group B. The mean aPPT was lower in group A than in group B, without statistical significance. The mean value of serum glucose is higher in group B (*p* = 0.386), but the maximum extreme value is higher in group A. 

There were no statistically significant differences between the two groups based on the average serum urea and creatinine readings. The liver function markers such as bilirubin, AST, and ALT did not show any statistically significant changes between the groups, and the average albumin was statistically significantly lower, in group A. We note higher maximum values of ALT and AST in group A, suggesting the importance of hepatocytolysis, which can anticipate a severe evolution of COVID-19. The mean values of serum amylase has shown no differences between the two studied groups.

The average value of creatine kinase is significantly increased in group A compared to group B (*p* = 0.016), indicating a higher proportion of cases with a severe evolution of COVID-19 in the group of patients with a history of cardiovascular disease. The average troponin I value between the two groups did not show any statistically significant differences. The average blood potassium, sodium, and chlorine levels did not differ statistically between the two studied groups.

Regarding the prevalence of comorbidities in the study group, it should be noted that, due to the exclusion criteria, oncology patients were not included in this study. Metabolic comorbidities, namely diabetes and obesity, predominated, with a higher percentage in group A (58.02%), but without statistically significant difference, compared to group B (48.14%). Neurological, renal, hepatic, and pulmonary comorbidities were present in a small number of patients in both groups.

Cardiovascular comorbidities had the highest prevalence in the total group among all comorbidities, suggesting a correlation between their presence and the prognosis of the groups with or without cardiovascular comorbidities.

Antiviral treatment with Remdesivir was administered to patients in both groups, while immunomodulation was carried out using tocilizumab or corticosteroids.

There was a statistically significant difference (*p* = 0.046) in the percentage of patients who died in group A, with a rate of 71.6%, compared to group B, where the percentage was 48.1%.

The summary of the results of this study includes statistically significant differences between patients with cardiovascular comorbidities experiencing severe forms of COVID-19 compared to those with other comorbidities:

- They have a higher risk of death.
- They have a younger average age and a shorter duration of hospitalization, which actually implies forms with rapidly severe evolutions in younger patients.
- The inflammatory profile, especially the C-reactive protein, is higher upon admission to the ICU.
- LDH and serum creatine kinase on admission to the ICU have higher mean values.

## 4. Discussion

We investigated the impact of cardiac comorbidities on mortality in severe forms of COVID-19 and found a statistically significant connection between cardiac damage and mortality in COVID-19 patients. In conclusion, a pre-existing cardiac condition, in the context of a severe form of a systemic disease that also includes cardiac tropism, can worsen the patient’s prognosis.

Understanding the pathophysiology of cardiovascular lesions in COVID-19 will significantly improve the treatment algorithm for patients with this condition and, implicitly, their prognosis.

Pathophysiological mechanisms of COVID-19 cardiac injury include the following:

A. Direct viral aggression

Isolation of SARS-CoV-2 from the heart of a deceased COVID-19 patient, with viral genome replication identified in the myocardium [7]. However, in situ hybridization demonstrated that the virus was present in myocardial interstitial cells without being confirmed in myocytes.

The histological lesions described during the autopsy included limited interstitial fibrosis and mononuclear inflammatory infiltrates in the heart, with evidence of the macrophages (CD68) and T-cells (CD4) [8]. These lesions did not meet the Dallas criteria for myocarditis [9]. Thus, the severity of cardiac damage in COVID-19 could not be correlated with the extensive myocardial cell damage or massive monocytic inflammatory infiltrates. Instead, many studies insist on intracardiac endothelial lesions observed by electron microscopy (EM) and immunohistochemistry (IHC). Evidence had shown the presence of severe lymphocyticendotheliitis in the heart, along with thrombotic lesions of small vessels and myocardial infarction [9].

Intra-COVID-19 myocarditis is characterized by diffuse ST-segment elevation, an increase in troponin T and NT-proBNP, decreased ejection fraction, myopericarditis on cardiac MRI, and the absence of obstructive lesions on coronary angiography [10,11].

B. Hyperactivation of the immune system-cytokine storm

Severe cases of COVID-19 are accompanied by massive releases of inflammatory mediators, including interleukins and tumor necrosis factors [12,13]. These cytokines contribute to the infiltration of immune cells into the myocardium, causing inflammation and potentially leading to myocardial damage [14].

This cytokine storm can affect the heart, similar to the immune system’s activation, which has been demonstrated to occurin sepsis with heart failure [15]. In COVID-19 patients, the modification of the immune system with dexamethasone improves their course [16].

C. Immunological disorders and autoimmunity

SARS-CoV-2 infection has been linked to immunological dysregulation, which in turn has been linked to autoimmune reactions against cardiac tissues. Research has indicated that COVID-19 individuals with myocardial problems have autoantibodies against cardiac antigens. This points to a possible autoimmune component that fuels inflammation and cardiac injury [17].

An interesting feature of COVID-19’s immunological response is the potential for molecular mimicry, in which viral proteins share structural similarities with host tissues, including the components of the myocardium. This similarity can trigger an autoimmune response, in which the immune system, activated to target the virus, can attack and damage heart cells [18].

D. Hypoxia

Respiratory failure, hypoxemia, hypotension, and shock lead to insufficient oxygenation of the myocardium, particularly affecting patients with a history of coronary artery disease or heart failure [19,20].

E. Blood clotting disorders

SARS-CoV-2 has been associated with a pronounced state of hypercoagulability. This hypercoagulability is characterized by an increased tendency of the blood to form clots, affecting both the microvasculature and large vessels. The virus triggers a complex cascade involving endothelial dysfunction, platelet activation, and coagulation system dysregulation, collectively contributing to a prothrombotic environment [21], leading to the formation of blood clots. Endothelial dysfunction is also accompanied by vasoconstriction, turbulent blood flow, and impaired fibrinolysis, which can also destabilize atheroma plaques [22]. Impacting the heart, it can lead to thrombosis in the coronary arteries, compromising the blood supply to the myocardium [23].

F. Dyselectrolytemias

Dysregulation of the renin–angiotensin–aldosterone system (RAAS) has been observed in COVID-19, with the virus interacting with ACE2 receptors, leading to ACE2 downregulation. This disruption of the ACE2 function can lead to imbalances in RAAS, impacting the electrolyte levels such as sodium and potassium [24]. Kidney damage in severe cases of COVID-19 may also play a role in the electrolyte imbalance that results in hyponatremia or hyperkalemia [25]. 

From moderate to severe COVID-19 infections, high temperature and sweating can lead to dehydration and further dyselectrolytemia [26]. The presence of symptoms such as nausea, vomiting, and diarrhea found in COVID-19 individuals with gastrointestinal involvement can lead to the significant loss of fluids and electrolytes, and consequent imbalances such as hypokalemia or metabolic acidosis [27]. The use of diuretics in high doses may contribute to electrolyte imbalances [28]. These electrolyte imbalances may be accompanied by cardiac arrhythmias or neurological complications [29].

G. Drug toxicity

For example, administration of azithromycin may result in adverse effects such as palpitations (≥1/1000, less than 1/100), and with a frequency that cannot be estimated from the available data: torsades de pointes (TdP), arrhythmias, including ventricular tachycardia, and QT prolongation [30].

H. Neurohormonal activation

SARS-CoV-2 infection triggers neurohormonal activation, including an increase in sympathetic activity and the release of stress hormones. Research by Borovoc et al. (2020) and Hartupee et al. (2020) demonstrated the link between neurohormonal activation and adverse cardiac outcomes, including arrhythmias and heart failure. The sustained activation of the sympathetic nervous system may contribute to stress and myocardial dysfunction [31,32]. 

Fox and Vander Heide et al. [33] devised the pathophysiological mechanisms favoring the occurrence of myocardial lesions. The authors proposed that both pulmonary microvascular lesions and hypoxia can lead to myocyte necrosis and right-heart stress. This can be supported by localized microvascular effects, since endotheliitis [34] can be associated with microthrombi, hemostasis, as well as changes in the renin–angiotensin system [35].

In the Woodruff R.C. study, 8460 patients with COVID-19 infection were evaluated. 11.4% (95%CI: 10.1–12.9%) of the patients presented a cardiovascular event during hospitalization. The rate of occurrence of these complications was significantly higher in patients with cardiovascular antecedents with a percentage of 23.4% (95%CI: 20.7–26.3%), compared to 6.2% of those without a history of cardiac diseases (95%CI: 5.1–7.6%) [36].

In the Woodruff R.C. study, the most common pre-existing pathologies were diabetes in 45%, hypertension in 78.2%, underlying cardiac disease in 61.9%, and chronic kidney disease in 30% [36]. Potentially immunosuppressive comorbidities increase the risk of severe infections [37,38,39]. In our study, metabolic complications included diabetes and obesity, representing a higher percentage of 58.02% in the group with a history of cardiovascular diseases compared to the group with no history of cardiovascular diseases, with a percentage of 48.14%, without a statistically significant difference. From the stand point of cardiovascular comorbidities, the most common pathologies were congestive heart failure in 44%, atrial fibrillation in 23.5%, and coronary heart disease in 28.4% [36]. In our study cardiovascular comorbidities included hypertension in 23 patients (28.39%), atrial fibrillation in 4 patients (4.93%), valvulopathies in 2 patients (2.49%), conduction disorders in 6 patients (7.40%), chronic ischemic cardiomyopathy in 41 patients (50.61%), and heart failure in 38 patients (46.91%).

In another study, Shi S et al. [40] found out that cardiac damage is a common consequence (19.7%) and was associated with an unexpectedly high risk of death while hospitalized. Furthermore, individuals with pre-existing cardiovascular disorders may be more vulnerable to COVID-19-induced heart injury, since around 30% and 60% of patients with cardiac injury in the current study had a history of coronary heart disease and hypertension, respectively, which were substantially more prevalent than in those without cardiac injury [40].

Similarly, a recent study [41] found that 25% and 58.3% of patients who were critically ill with COVID-19 had underlying cardiac disease and hypertension. According to the ‘Diagnosis and Treatment of Novel Coronavirus Pneumonia (Trial Version 4)’ [42], elderly persons with underlying conditions are more likely to be infected with SARS-CoV-2 and become extremely unwell, particularly those with hypertension, coronary heart disease, and diabetes. In contrast, acute inflammatory reactions can cause ischemia in the presence of pre-existing cardiovascular disorders. During the systemic inflammatory response, the inflammatory activity within coronary atherosclerotic plaques increases, making them more likely to rupture [43].

Inflammation also induces endothelial dysfunction and increases blood procoagulant activity, which can help build an occlusive thrombus over a ruptured coronary plaque [44]. Based on these findings, the authors proposed that a strong inflammatory response combined with pre-existing cardiovascular illness may precipitate the cardiac damage seen in COVID-19 patients [40].

In our study, the mean troponin was above the normal value in both groups. Elevated serum troponin values are often found in clinical practice in the COVID-19 patient, suggesting significant myocardial damage [45,46]. 

Bois et al. [47] seem to support the idea of microthrombi occurring in association with COVID-19. In a group of 15 post mortem patients, the authors observed that fibrin microthrombi were more common, at 80%, compared to acute ischemic injury at 13% and myocarditis at 33%. This suggests that the appearance of microthrombi is one of the factors that accentuate myocardial damage [45,47]. 

Clinical studies note that in critical cases the average level of D-dimers is 2.4 mg/L, compared to mild cases where the average level is 0.5 mg/L [48]. In other series of cases, D-dimers values > 3 mg/L were noticed in 85% of the patients who died post-COVID-19 [49], while other studies found D-dimers values > 1 mg/L in 81% of the patients who did not survive COVID-19, and only 24% of survivors had D-dimers > 1 mg/L [50,51,52].

In a group study, the coagulation status and the average D-dimer level was higher in the group with a history of cardiovascular diseases but without a statistically significant difference compared to the group with no history of cardiovascular diseases. The meaning of aPPT was without statistical significance, and it was lower in group A than group B.

In our study, the group of patients with cardiovascular disease presented higher average values of inflammatory factors (statistically significant for C-reactive protein), which correlates with the possibility of more severe forms of COVID-19 and a higher percentage of death.

The role of biomarkers in the early diagnosis of these patients is crucial in determining their prognosis. C-reactive protein, leukocytes, albumin, and ferritin are considered predictors of mortality [53,54].

However, a study conducted by Iturbide-Mauricio L et al. on patients with COVID-19 observed an unfavorable evolution in those who received corticosteroid therapy and invasive mechanical ventilation [55].

K.L. Sandoval-Bedolla et al. studied another predictive factor for mortality in COVID-19 patients. They calculated the systemic immune inflammation index (SII) as neutrophils × platelets/lymphocytes and showed that the SII is a simple, effective, and predictive measure of death in hospitalized COVID-19 patients, which can be used as a prognostic indicator for patients with COVID-19 [56].

The upregulation of miR-208 has been linked to a higher proinflammatory and prothrombotic state in the context of severe SARS-CoV-2 infection, which increases the risk of ischemic cardiovascular manifestations, including early ST-elevation myocardial infarction in COVID-19 patients [57,58,59]. The possible impact of SARS-CoV-2 on human-expressed miRNAs is noteworthy. Preliminary research using the SARS-CoV-2 genome’s sponge effect revealed a relationship between miRNA-20a-5p and severe disease manifestation linked to clinical deep vein thrombosis and cardiovascular symptoms [60]. This fresh approach to miRNA research may prove to be a useful instrument in improving our comprehension of the origins of COVID-19 and its various clinical manifestations, as well as in creating more precise antiviral treatments [61].

The limitations of our study start from the relatively small size of the patient group, which does not allow for strong conclusions. Additionally, the study was conducted at the beginning of the pandemic, on patients with severe forms of COVID-19 that required a multidisciplinary approach and complex therapies. Clinical practice defined biomarkers useful in predicting severe forms and personalized treatment algorithms towards the end of the pandemic. Furthermore, access to investigations and therapies requiring prolonged exposure to aerosols with SARS CoV-2 was limited at the beginning of the pandemic and gradually improved after the introduction of vaccination.

## 5. Conclusions

Our study provides additional evidence that supports the idea that patients with pre-existing cardiovascular diseases who experience severe forms of COVID-19 have a higher risk of death, a younger average age, faster progression towards death, and higher inflammatory markers than patients with other types of comorbidities.

Cardiovascular comorbidities in COVID-19 worsen the prognosis of patients and their management must include, in addition to antiviral and anticoagulant treatment, personalized cardiac therapy capable of improving patient outcomes.

## Figures and Tables

**Table 1:** The comparative values of patients with severe COVID-19 and a history of cardiovascular disease, with characteristics versus patients with severe COVID-19 and no history of cardiovascular disease, with characteristics.

	Severe COVID-19 with a History of Cardiovascular Disease Group A (N 1 = 81)			Severe COVID-19 with No History of Cardiovascular Disease Group B (N 2 = 27)			*p* (T-t)
	Mean	SD	Min; Max	Mean	SD	Min; Max	
Age (years old)	68.57	13.23	21; 99	69.44	17.69	20; 91	<0.001
Intensive care unit Length of stay (days)	4.98	4.57	1; 25	7.78	6.25	1; 25	<0.001
SpO_2_ upon admission (%)	71.32	65.43	48; 90	76.82	71.54	61; 90	0.712
Leucocytes (× 10^9^/L)	12.74	7.49	0; 48.05	13.86	8.85	2.81; 40.49	0.527
ESR (mm/1 h)	80.06	38.57	3; 178	70.18	42.14	10; 140	0.264
C-reactive protein mg/L	127.15	84.87	3; 425	69.01	43.25	6; 192	<0.001
LDH μmol/sl	1510.43	885.77	2; 58,855	1038.62	983.80	4; 3529	0.022
D-dimer mcg/mL	3.74	1.39	0; 7.76	3.34	0.78	2.34; 4.78	0.064
Activated partial thromboplastin time—aPTT seconds	34.70	14.51	0; 114.40	42.17	21.23	19.10; 110	0.098
Serum glucose mg/dL	167.35	94.04	37; 516	186.14	106.21	52; 432	0.386
Serum urea mg/dL	97.19	79.22	20; 400	81.59	57.80	28; 232	0.348
Serum creatinine mg/dL	1.96	1.91	0.64; 11.6	1.68	1.65	0.65; 7.7	0.497
ALT U/L	45.16	234	5; 2321	30.87	24.77	7; 162.98	0.361
AST U/L	53.45	136	15; 1328	30.97	17.31	14; 130.98	0.081
Serum total bilirubin mg/dL	1.04	1.65	0.24; 14.16	0.68	0.44	0.22; 2.20	0.262
Serum direct bilirubin mg/dL	0.51	1.15	0.08; 9.84	0.36	0.23	0.11; 1.23	0.503
Serum albumin g/dL	4.04	1.01	1.8; 5.9	4.78	0.73	2.99; 6	0.007
Serum amylase U/L	70.83	42.25	11; 200	66.18	56.68	8; 280	0.651
Serum creatine kinase U/L	533.82	520.21	37; 7869	201.40	265.99	43; 1318	0.016
Serum troponin ng/dL	0.29	0.58	0.02; 2.49	0.34	0.63	0.05; 2.99	0.706
Serum sodium mmol/L	141.01	9.44	111; 117	140.18	6.12	129; 157	0.602
Serum potassium mmol/L	4.74	4.80	2.2; 47	3.99	0.60	2.60; 5.80	0.425
Serum chlorine mmol/L	103.85	12.05	28.2; 136.9	102.90	6.39	89.2; 114.3	0.695

Abbreviations and normal values: SD = standard deviation; Max = maximum value; Min = minimum value; T-t = student test (t) for differences between means; Leucocytes—5000–10,000/μL; Erythrocytes sedimentation rate (ESR)—0–15 mm/1 h; C-reactive protein—0–1 mg/L; Lactate dehydrogenase—0–4.2 μmol/sl; D—dimer-0–0.5 μg/mL; aPTT < 40 s; Serum glucose—60–99 mg/dL; Serum urea 18–55 mg/dL; Serum creatinine 0.8–1.3 mg/dL; ALT, alanine-aminotransferase 0–45 U/L; AST, aspartate-aminotransferase—0–35 U/L; Total bilirubin—0.1–1.2 mg/dL; Direct bilirubin—0–0.2 mg/dL; serum amylase < 100 U/L; Serum creatine kinase < 308 U/L; Serum albumin—3.5–5.2 g/dL; Serum troponine—0.01 ng/dL; Serum sodium-136–145 mmol/L; Serum potassium—3.3–5.1 mmol/L; Serum chloride—94–110 mmol/L.

**Table 2:** Categorial variables characteristics of the patients with severe COVID-19 with a history of cardiovascular disease, compared to those of the patients with severe COVID-19 with no history of cardiovascular disease.

	Severe COVID-19 with a History of Cardiovascular Disease (N 1 = 81)		Severe COVID-19 with No History of Cardiovascular Disease (N 2 = 27)		Significance Level Χ^2^ Test *p*
	N	%	n	%	
Sex Female Male	33 48	40.7 59.3	13 14	48.1 51.9	0.653
Metabolic comorbidities	47	58.02	13	48.14	0.502
Neurological comorbidities	15	18.51	8	29.62	0.342
Renal and hepatic comorbidities	2	2.46	5	18.51	0.013
Pulmonary comorbidities	5	6.17	3	11.11	0.671
Deaths	58	71.6	13	48.1	0.046

## Data Availability

Data Availability Statements are available upon request, through the corresponding author.
